# Pandemie und Wertewandel? Verschiebungen von Wertprioritäten in der österreichischen Bevölkerung und in einzelnen Wähler*innenschichten als Reaktion auf die COVID-19-Pandemie

**DOI:** 10.1007/s11614-022-00505-z

**Published:** 2022-10-14

**Authors:** Anja Eder, Wolfgang Aschauer, Franz Höllinger, Martin Ulrich

**Affiliations:** 1 Institut für Soziologie, Universität Graz, Universitätsstraße 15, 8010 Graz, Österreich; 2 Abteilung Soziologie und Kulturwissenschaft, Paris-Lodron-Universität Salzburg, Kapitelgasse 4–6, 5020 Salzburg, Österreich

**Keywords:** Konservatismus, Konformität, Corona-Krise, Values-in-Crisis-Studie, Wertewandel, Conservatism, Conformity, Corona crisis, Values in Crisis Study, Value change

## Abstract

Soziologische Zeitdiagnosen suggerieren, dass tiefgreifende gesellschaftliche Krisen wie die COVID-19-Pandemie unsere Wertorientierungen infrage stellen und auch relativ kurzfristig ändern könn(t)en. Von dieser Beobachtung ausgehend, wird untersucht, ob es in Österreich im Zeitraum Mai 2020 bis März/April 2021 zu signifikanten Verschiebungen von Wertprioritäten nach der Skala von Shalom Schwartz kam. Als Datenmaterial dienen die beiden ersten Wellen der Panelstudie Values in Crisis. Für die Interpretation der Ergebnisse sind zwei theoretische Annahmen zentral: erstens die These eines zunehmenden Konservatismus und zweitens die These der Wirkmacht politischer Diskurse in Zeiten des (wieder)aufkeimenden Populismus. Besondere Berücksichtigung findet im Beitrag zudem eine methodologische Auseinandersetzung mit dem Wandel der Bedeutung von Fragebogenitems aufgrund der COVID-19-Pandemie.

Die empirischen Analysen bestätigen – entgegen einigen aktuellen Zeitdiagnosen – eine deutliche Stabilität von Wertorientierungen. Verändert hat sich vor allem der Wert der Konformität, indem er für einen Teil der Bevölkerung wichtiger wurde; gleichzeitig verlor der Wunsch nach einer hedonistischen Lebensweise etwas an Bedeutung. Konformität wurde insbesondere für die Wähler*innen der Regierungsparteien wichtiger, während sich dieser Trend vor allem bei den Wähler*innen der FPÖ nicht zeigte. Da die beobachteten Verschiebungen von Wertprioritäten vor allem „pandemie-sensible“ Wertedimensionen betreffen, lässt sich auf der Basis der vorliegenden Ergebnisse insgesamt eher von einer kurzfristigen Reaktion auf die Krise und weniger von einem längerfristigen Wertewandel ausgehen.

## Abstract (en)

Recent sociological diagnoses suggest that profound social crises such as the COVID-19 pandemic challenge our value orientations and could change them even in the relatively short term. Based on this observation, we investigate whether significant shifts in value priorities according to the Shalom Schwartz scale took place in Austria in the period May 2020 to March/April 2021. The first two waves of the Values in Crisis panel study serve as data material. Two theoretical assumptions are central to the interpretation of the results: first, the thesis of a trend toward conservatism and second, the thesis of the effective power of political discourses in times of (re)emerging populism. The article also pays special attention to a methodological discussion of changes in the meaning of questionnaire items due to the COVID-19 pandemic.

The empirical analyses confirm a clear stability of value orientations. Above all, the value of conformity has changed, becoming more important for a significant part of the population; at the same time, the desire for a hedonistic lifestyle lost some of its importance. Conformity became more important, particularly for voters of the governing political parties, while this trend was not apparent, especially among voters of the FPÖ. Since the observed shift in value priorities mainly concerns “pandemic-sensitive” value dimensions, the results suggest a short-term reaction to the crisis rather than a long-term change in values.

## Einleitung

In der soziologischen Wertewandelsforschung findet man unterschiedliche, zum Teil einander widersprechende Annahmen zur Frage der Stabilität und Veränderbarkeit von Wertvorstellungen. Einerseits wird davon ausgegangen, dass Werte im Zeitverlauf relativ stabil und von situativen Einflüssen weitgehend unberührt sind. Die grundlegenden Wertorientierungen, die sich Menschen in ihrer Jugend und im frühen Erwachsenenleben aneignen, ändern sich demnach im weiteren Verlauf ihres Lebens in der Regel nur wenig oder allenfalls längerfristig (Scherer und Roßteutscher 2019; Rudnev et al. 2016). Zum anderen wurden bisherige Werteveränderungen sowohl langfristigen Modernisierungsprozessen und gesellschaftlichen Umbrüchen als auch kurzfristigen Krisen zugeschrieben (Klein und Pötschke 2004, S. 443 ff.; Welzel 2009, S. 110 f.).

Tiefgreifende gesellschaftliche Krisen wie die COVID-19-Pandemie lösen starke Unsicherheiten, Irritationen und Bedrohungsgefühle aus, die unsere Weltanschauungen und Wertorientierungen infrage stellen und unsere Vorstellungen davon, worauf es im Leben ankommt, auch relativ kurzfristig ändern können (Luhmann 1994 [1971], S. 16; Schwartz 2005, S. 17). Dies suggerieren auch verschiedene soziologische Zeitdiagnosen: So erwartete etwa Heinz Bude^1^ zu Beginn der Krise eine Stärkung des gesellschaftlichen Zusammenhalts infolge der kollektiv erlebten Verwundbarkeit. Jutta Allmendinger^2^ konstatierte vor allem im Hinblick auf die Rollenverteilung in Partnerschaften eine Renaissance konservativer Werthaltungen. Insgesamt legen aktuelle Studien nahe, dass die Mehrfachkrisen der letzten Dekade insbesondere zu einem Wiedererstarken konservativer Werte beitragen würden (Strobl 2021; Voicu et al. 2016). Demnach wäre zu erwarten, dass sich der Trend zum Wiederaufleben bewahrender Werte (wie Sicherheit, Tradition und Konformität) im Zuge der COVID-19-Pandemie fortsetzt oder sogar verstärkt (Aschauer et al. 2022). Vor diesem Hintergrund gehen wir im vorliegenden Beitrag der Frage nach, inwieweit es zu Verschiebungen der Priorität der zehn Grundwerte des Wertemodells von Shalom Schwartz (1992) im Verlauf der Krise kam.

Da die Regierungs- und Oppositionsparteien im Zuge der COVID-19-Pandemie konträre und sich verändernde Positionen vertraten, liegt ein besonderes Augenmerk des Beitrags auch auf potenziellen Verschiebungen von Wertprioritäten der Wähler*innen einzelner Parteien. Zu Beginn der Pandemie sprachen die beiden Regierungsparteien ÖVP und Grüne in Bezug auf die pandemierelevanten Herausforderungen oft mit einer Stimme.^3^ In diesem anfänglichen Ausnahmezustand zeigten sich große Teile der österreichischen Bevölkerung mit den Maßnahmen zur Pandemiebekämpfung einverstanden und die Regierung genoss ein hohes Vertrauen, was auch als „rally around the flag“-Phänomen bekannt ist (Partheymüller et al. 2020). Die vordergründige Einigkeit zwischen den Parteien und deren Wähler*innen schien jedoch rasch wieder zu verblassen. Seit Ende März 2020 sank das Vertrauen in die Regierung kontinuierlich; die FPÖ machte durchgängig gegen die staatlichen Corona-Maßnahmen Stimmung. Nach einer Schockstarre in den Anfangsmonaten der Pandemie wurden allmählich die sozialen, wirtschaftlichen und psychischen Folgen der Pandemiebekämpfungsmaßnahmen absehbar (siehe Dörre 2020; Foissner et al. 2021). Unterschiedliche Vorstellungen hinsichtlich des Spannungsverhältnisses zwischen der Freiheit und der Sicherheit der Bürger*innen wurden sodann zur andauernden Streitfrage.

Für die Wertewandelsforschung stellen diese Kontroversen ein wichtiges Untersuchungsfeld dar. Die Corona-Pandemie liefert die seltene Gelegenheit zu beobachten, ob und wie sich die Wertorientierungen der Österreicher*innen und spezifischer Wähler*innengruppen als Reaktion auf akute Krisen potenziell verändern. Anhand von Befragungsdaten der österreichischen Values-in-Crisis-Studie (VIC 2020 und VIC 2021; Aschauer et al. 2021) gehen wir im vorliegenden Beitrag deshalb der Frage nach, ob sich im Zuge der COVID-19-Pandemie signifikante Werteverschiebungen in der österreichischen Gesellschaft im Allgemeinen und im politischen Spektrum Österreichs im Besonderen beobachten lassen. In dieser Online-Panel-Studie, an der mehr als 2000 Österreicher*innen teilnahmen, wurde die 21-Item-Version des Portraits Values Questionnaire von Schwartz et al. (2001; Schmidt et al. 2007) implementiert, um Veränderungen in mehreren grundlegenden Wertorientierungen präzise messen zu können. Zusammenfassend lauten unsere Forschungsfragen:

1. Wie haben sich die Wertorientierungen der Österreicher*innen im Verlauf der Pandemie (von Mai 2020 bis März/April 2021) verändert?
2. Welche Werte sind den Wähler*innen unterschiedlicher Parteien besonders wichtig?
3. *Kam es im Verlauf der Pandemie zu Werteverschiebungen nach Parteipräferenz?*

Bereits an dieser Stelle sei darauf hingewiesen, dass einzelne Frageformulierungen des etablierten Portraits Values Questionnaire von Schwartz (siehe Tab. 3 im Anhang) im Kontext der COVID-19-Pandemie zu unterschiedlichen Urteilen der Befragten führen können. Aspekte der Sicherheit und Konformität erlangen in Zeiten der Bedrohung des Gesundheitssystems und staatlich verordneter Freiheitseinschränkungen wie Lockdowns, Kontaktbeschränkungen und Maskenpflicht eine „coronaspezifische Bedeutung“ für Befragte und könnten zum Beispiel zu einer coronabedingten Abnahme der Wertschätzung von Hedonismus und Selbstbestimmung führen. In den folgenden Analysen der Veränderungen von Wertprioritäten soll daher ein besonderes Augenmerk darauf gelegt werden, inwieweit beobachtbare Werteverschiebungen auf ein unterschiedliches Werteverständnis in Zeiten der Pandemie zurückgeführt werden können.

## Theoretischer Hintergrund

Eine der bekanntesten Konzeptionen von Werten ist jene von Shalom H. Schwartz (1992; Schwartz et al. 2001). Werte sind für ihn situationsübergreifende Überzeugungen und Zielvorstellungen (Schwartz 1992, S. 4; Schwartz et al. 2010, S. 422), die von Individuen, Gruppen oder ganzen Gesellschaften vertreten werden (Haller und Müller Kmet 2019, S. 51; Rudi 2009, S. 609; Welzel 2009, S. 109). Sie stellen einen grundlegenden Orientierungsrahmen für Einstellungen und Handlungen dar und können auch wesentliche Einflussfaktoren für die Übernahme von Ideologien sein (Schwartz et al. 2010).

**Figure 1:**
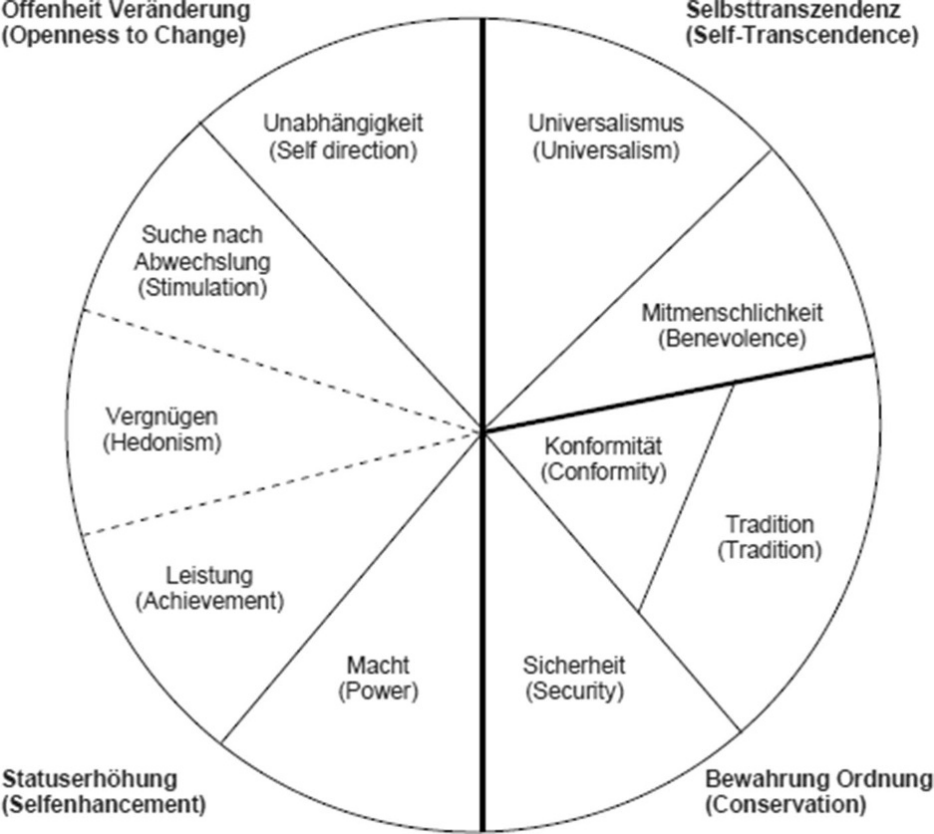

Schwartz (1992) unterscheidet in einem kreisförmigen Modell zehn Wertorientierungen, die grundlegende Wertedispositionen der Individuen markieren (siehe Abb. 1). Diese lassen sich zwei übergeordneten Dimensionen zuordnen. Während Selbstbestimmung, Stimulation und Hedonismus eine stärkere Bereitschaft für Veränderung implizieren, bilden Konformität, Tradition und Sicherheit den gegenüberliegenden Pol der bewahrenden Werte. Relativ unabhängig davon streben Individuen entweder nach Macht und Leistung oder treten mit Mitmenschlichkeit und Universalismus für ein tolerantes Miteinander und für eine Gleichberechtigung der Individuen ein (Schwartz 1992, S. 4, S. 14 f., S. 42 ff.; Schwartz et al. 2010, S. 424 ff.) Diese zehn Werte können so auch in vier Überkategorien zusammengefasst werden (siehe Abb. 1).

Gemessen werden diese Wertorientierungen im Portraits Values Questionnaire anhand von Kurzportraits der Wertprioritäten von fiktiven Personen. Befragte geben bei diesen an, ob sie der Person ähneln oder in ihren Wertespektren stark abweichen (siehe Tab. 3 im Anhang). Mit dieser Methode gelingt es nach Schwartz et al. (2001), soziale Erwünschtheit in der Messung von Werthaltungen zu reduzieren; zudem eignen sich die eingängigen Portraits besonders gut für bevölkerungsrepräsentative Messungen.

### Verschiebung von Wertprioritäten in Krisenzeiten

Die empirische Werteforschung hat gezeigt, dass gesellschaftliche Transformationen wie jene eines Wertewandels üblicherweise sehr langsam stattfinden, zumal grundlegende Wertvorstellungen historisch gewachsen sind (für Europa Rudnev et al. 2016). Bisherige Werteveränderungen wurden sowohl langfristigen Modernisierungsprozessen als auch gesellschaftlichen Umbrüchen und kurzfristigen Krisen zugeschrieben (Klein und Pötschke 2004, S. 443 ff.; Welzel 2009, S. 110 f.; Aschauer et al. 2022, S. 213). Übereinstimmend legen Studien nahe, dass die Mehrfachkrisen der letzten Dekade vor allem förderlich für die Renaissance konservativer Werte zu sein scheinen (siehe z. B. im Überblick Voicu et al. 2016). Ähnlich führen auch Norris und Inglehart (2019) die gesellschaftlichen Entwicklungen des Brexit oder der Wahl von Donald Trump und des (wieder) stärker aufkeimenden Populismus auf einen generellen „cultural backlash“ zurück. Sie meinen damit, dass sich in zahlreichen westlichen Gesellschaften eine Rückbesinnung auf traditionelle Werte bzw. eine aktive Gegenbewegung gegenüber postmateriellen Werten beobachten lässt. Auch Natascha Strobl (2021) stellt für Österreich und andere Länder jüngst fest, dass die sich überlagernden und sich verstärkenden Mehrfachkrisen der letzten Jahre (wie die globale Finanz- und Wirtschaftskrise, die Fluchtbewegungen im Jahr 2015 sowie aktuell die Corona-Pandemie) dazu beigetragen haben, dass konservative Parteien einen neuen „radikalisierten Konservatismus“ vertreten, mit dem sie ganz unterschiedliche Wähler*innenschichten erfolgreich ansprechen.

Wenn wir nun davon ausgehen, dass sich durch die COVID-19-Pandemie im Zeitraum Mai 2020 bis März/April 2021 bestimmte Wertprioritäten verändert haben (könnten), dann erscheinen uns folgende Entwicklungen plausibel: (1.) Angesichts der Ausnahmesituation und staatlich verordneten Einschränkungen der Sozialkontakte lässt sich annehmen, dass Werte wie Stimulation, Hedonismus und der Wunsch nach Selbstbestimmung (vorübergehend) an Bedeutung verlieren; auch der Wert der Leistung könnte durch die wiederholten Lockdowns im Verlauf der Pandemie weniger wichtig geworden sein. (2.) Da die Pandemie in breiten Teilen der Bevölkerung Gefühle der Unsicherheit und Bedrohung verstärkt hat, erscheint es plausibel, dass das Bedürfnis nach Sicherheit und Konformität mit den staatlichen Maßnahmen in der Bevölkerungsmehrheit steigt. (3.) In der ersten Phase der Pandemie wurde von einigen Beobachter*innen eine verstärkte Bereitschaft zur Solidarität und Unterstützung anderer festgestellt. Daher ist es naheliegend, dass der Wert der Mitmenschlichkeit im Verlauf der Krise seine hohe Bedeutung beibehält, oder aber auch, dass dieser Wert nach dem Abflauen der ersten Solidaritätswelle mit zunehmender Dauer der Krise an Bedeutung verliert.

Kommt es bei einzelnen Wertdimensionen zu Veränderungen, wirft dies die Frage auf, ob es sich um eine Fortsetzung eines längerfristig anhaltenden Trends eines Wertewandels oder um temporäre Verschiebungen von Wertprioritäten handelt. Letztere könnten nicht nur auf die Ausnahmesituation der Pandemie zurückzuführen sein, sondern auch auf eine „coronaspezifische Konnotation“ bestimmter Formulierungen der Schwartz Werte-Items (siehe Tab. 3 im Anhang). In der Ablehnung des Hedonismus-Items „ich bin ein Mensch, der jede Gelegenheit nutzt, um Spaß zu haben“ spiegelt sich womöglich wider, dass Spaßhaben während der Pandemie als sozial unerwünscht galt, und nicht, dass jemand weniger hedonistisch geworden ist. Es ist auch anzunehmen, dass die vorgegebenen Werteportraits zu Sicherheit und Konformität im Zuge der Pandemie vor allem im Kontext des staatlichen Gesundheitsschutzes und der Einhaltung der staatlich verordneten Corona-Regeln interpretiert werden. Speziell der Wert der Konformität wird mit den beiden Items „Befolgung von Regeln“ und „ein anständiges Leben führen“ erhoben, was im Kontext der Corona-Pandemie mit der Konformität gegenüber den staatlichen Corona-Maßnahmen assoziiert werden dürfte. Die Frage, ob es sich um einen tatsächlichen Wertewandel oder um eine temporäre Prioritätenverschiebung handelt, lässt sich nur durch Panelbefragungen über einen längeren Zeitraum hinweg beantworten. Wir möchten jedoch versuchen, durch Detailanalysen einzelner Wertedimensionen erste Hinweise auf diese Frage zu gewinnen.

### Politische Diskurse rund um party cues und Populismus

In breiten Teilen der Bevölkerung scheint immer häufiger der Eindruck vorzuherrschen, dass die Politik nicht in der Lage sei, die Krisen und Herausforderungen der Gegenwart zu lösen, wodurch sie oftmals als „hinterherhinkende oder sich durchwurstelnde Feuerwehr“ (Rosa 2016, S. 376) wahrgenommen wird. Auch weil aus Sicht der Bevölkerung langfristige Visionen in der Politik fehlen und das politische Krisenmanagement oft als simples Reagieren auf strukturelle Anpassungszwänge aufgefasst wird, steigen die politische Entfremdung und Politikverdrossenheit (z. B. Huth 2004; für Österreich siehe Eder et al. 2020). Hinzu kommt ein genereller Vertrauensverlust in staatliche Institutionen.^4^ Diese Entwicklungen hätten auch den Weg für rechtspopulistische Bewegungen geebnet, die von „pessimistischen Nostalgikern“^5^ angeführt werden und in einer Anti-Establishment-Attitude für sich beanspruchen, mit der Stimme des Volkes zu sprechen (Crouch 2021, S. 187 ff.).

Schwartz et al. (2010) haben nachgewiesen, dass Wertorientierungen sowohl mit politischen Grundhaltungen (z. B. „traditional morality“, „equality“, „blind patriotism“), als auch mit der Selbstplatzierung der Befragten auf dem politischen Links-Rechts-Spektrum und der Präferenz für linke oder rechte Parteien in Verbindung stehen. Damit beeinflussen grundlegende Wertorientierungen, welche politischen Konzepte vertreten werden (Schwartz et al. 2010, S. 422 ff.). *Wie* Werte von parteipolitischen Positionen beeinflusst werden können, erklärt der Politikwissenschafter Philipp E. Converse damit, dass sich die Bevölkerung aufgrund der Komplexität der sozialen Welt an Einstellungen von Personen, Gruppen und Organisationen orientiert, deren Urteil sie vertrauen (Converse 1975, S. 79 ff., S. 93 ff., 2006, S. 5 ff., S. 14 ff.). Politische Parteien senden ihre Positionen in Form von sogenannten *cues* an die Bevölkerung aus, die helfen, sich politisch zu positionieren (Bakker et al. 2020, S. 1061 ff.; Kam 2005, S. 165, S. 170 ff.). Individuen, die sich verstärkt mit einer Partei identifizieren, sind umso empfänglicher für die *cues*, die diese Partei ihnen vermittelt. *Cues* werden auch genutzt, um sicherzustellen, dass sich Wähler*innen nach den Erwartungen der Partei positionieren. In diesem Fall erfüllen sie eine expressive Funktion und dienen dem Erhalt einer sozialen Identität (Bakker et al. 2020, S. 1061 ff., S. 1066 ff.).^6^

Nun war es zu Beginn der Pandemie für alle politischen Parteien von Bedeutung, ihre Position zum Krisenmanagement als *cues* an die tatsächlichen und potenziellen Unterstützer*innen zu vermitteln. Ziel war es, die breite Masse zum Befolgen von Corona-Maßnahmen oder zum Widerstand gegen diese zu mobilisieren. Obwohl *cue*-Effekte prinzipiell bei allen politischen Eliten eine Rolle spielen, dürfte ihr Einfluss bei populistischen Akteur*innen stärker sein. Sie inszenieren sich typischerweise als demokratische und „volks“nahe Opposition in einem System, das die politische Macht in illegitimen Händen oder Prozessen verortet, statt sie rechtmäßig der Bevölkerung zu übertragen. Aus dieser Sichtweise entsteht die Trennung zwischen einem vermeintlich homogenen „Volk“, dessen Willen in der Politik ohne Kompromiss durchgesetzt werden soll, und den Eliten, die diesem „Volk“ aus egoistischen und/oder böswilligen Gründen die politische Macht vorenthalten. Damit lehnt der Populismus die Pluralität und Konsensbildung typischer Demokratien ab (Rensmann 2006, S. 63 ff.; Mudde und Kaltwasser 2015, S. 498–506). Gesellschaftliche Krisen wie die Corona-Pandemie und politische Legitimationskrisen können nun zur Stärkung populistischer Akteur*innen beitragen (Decker 2006, S. 13 ff.; Mudde und Kaltwasser 2015, S. 495 ff.; Aschauer 2017, S. 308 ff.). Diese sprechen vor allem Personen an, die sich Modernisierungsprozessen nicht anpassen können oder wollen, indem sie auch Emotionen wie Wut oder Zorn zur Mobilisierung nutzen (Salmela und von Scheve 2018, S. 439 f.).

Zu den Eliten zählen für den Populismus neben Parteien oft auch andere Elemente des Establishments wie z. B. öffentlich-rechtliche Medien oder die Wissenschaft. Mede und Schäfer (2020) argumentieren, dass populistische Akteur*innen der Wissenschaft ihre epistemische Autorität absprechen, weil sie diese als Teil der Elite ansehen. Wissenschafter*innen wird vorgeworfen, durch Eliteninteressen korrumpierte Wissenschaft zu betreiben, die den gesunden Menschenverstand des „Volkes“ herabwürdige und delegitimiere. Da politische Entscheidungen auch auf wissenschaftlichen Erkenntnissen fußen, wird somit auch die politische Souveränität des „Volkes“ als bedroht wahrgenommen (Mede und Schäfer 2020, S. 480 ff.). Im Zuge der COVID-19-Pandemie wurden viele politische Maßnahmen auf Basis epidemiologischer und virologischer Studien verhängt, was von populistischen Akteur*innen zur Mobilisierung genutzt wurde. So fiel die rechtspopulistische Freiheitliche Partei Österreichs (FPÖ) dadurch auf, dass sie die Corona-Maßnahmen der Regierung als unverhältnismäßig und das Krisenmanagement als inkompetent und fehlgeleitet darstellte. Begründet wurde diese antagonistische Haltung gegenüber der Regierung und den staatlich verordneten Regeln mit der Sorge um die Freiheit des „Volkes“. Diese Haltung wurde medial in Form von *cues* verbreitet, was dazu führte, dass ihre Basis starken Nonkonformismus gegenüber den staatlich verordneten Regeln entwickelte und Schutzmaßnahmen oft nicht befolgte (Mellacher 2021).^7^

Im Gegensatz zur FPÖ hatten die Regierungsparteien ein Interesse daran, dass die von ihnen eingeführten Maßnahmen befolgt werden. Um dies zu erreichen, sandten sie *cues* an die Bevölkerung aus, die zur Konformität gegenüber den staatlichen Maßnahmen aufriefen. Eingefordert wurde der Konformismus mit der Begründung einer gesamtgesellschaftlichen Solidarität, die die Risikogruppen schützen und die Überbelastung des Gesundheitssystems verhindern solle. Neben deutlichen Unterschieden in der Wichtigkeit von Konformität zwischen Wähler*innen der Regierungsparteien und FPÖ gehen wir davon aus, dass sich die Wähler*innen der Regierungsparteien gerade zu Beginn der Pandemie in der Wertorientierung der Konformität stärker ähneln als Wähler*innen anderer Parteien und Nichtwähler*innen. Da die *cues* zur Konformität nur zu Beginn der Krise einheitlich waren und Grün-Wähler*innen ein non-konformeres Verhalten zugeschrieben wird^8^ (für Deutschland siehe Switek 2017), vermuten wir weiters, dass sich die Wähler*innen der Regierungsparteien nach einem Jahr Pandemie wieder stärker auseinanderentwickelt haben.

## Daten und Methoden

Für die empirischen Analysen wurde auf die beiden ersten Erhebungswellen der Online-Panelstudie „Values in Crisis“ (VIC) in Österreich zurückgegriffen (Aschauer et al. 2021). Die Stichprobenziehung erfolgte anhand von Quotenvorgaben, bei den meisten Merkmalen konnte eine gute Annäherung an die Gesamtbevölkerung erreicht werden.^9^ Unsere Analysen beziehen sich auf jene 995 Befragten, die an beiden Erhebungen – im Mai 2020 und im März/April 2021 – teilnahmen und bei denen aufgrund der Ausfülldauer von einer substanziellen Beantwortung der Schwartz-Werteskala auszugehen ist.^10^ Die longitudinale Analyse dieser Panelstichprobe ermöglicht es, intraindividuelle Werteverschiebungen in der Pandemie sowohl insgesamt als auch nach Parteizugehörigkeit zu untersuchen.

In einem ersten Schritt der Analyse werden die Veränderungen der Wichtigkeit der zehn Grundwerte zwischen den beiden Erhebungswellen (Abschn. 4.1) und die Unterschiede in den Wertepräferenzen nach Wähler*innengruppen im Zeitverlauf (Abschn. 4.2) näher analysiert. In der Wertemessung von Schwartz geben die Befragten auf einer 6‑stufigen Antwortskala an, wie ähnlich sie bestimmten Personenbeschreibungen sind (von „ist mir sehr ähnlich“ bis „ist mir überhaupt nicht ähnlich“)^11^. Die beiden Personenbeschreibungen hinsichtlich der Wertorientierung Konformität lauten etwa: „Er/Sie glaubt, dass Leute das machen sollten, was man ihnen sagt. Er/Sie meint, dass Leute sich immer und überall an Regeln halten sollten, selbst wenn es niemand sieht.“ und „Es ist ihm/ihr wichtig, ein anständiges Leben zu führen. Er/Sie möchte alles vermeiden, was Leute als Fehltritt bezeichnen könnten.“ (siehe alle Personenbeschreibungen in Tab. 3 im Anhang). Auf der Basis der subjektiven Einschätzungen der Ähnlichkeit zu den Personenbeschreibungen leitet Schwartz sodann die Wichtigkeit der jeweiligen Wertorientierungen ab.

In der vorliegenden Analyse wird die (relative) Wichtigkeit der zehn Grundwerte nach Schwartz anhand von zentrierten Mittelwerten untersucht.^12^ Der Mittelwertvergleich zeigt, dass beim Wert Konformität eine besonders starke Veränderung stattgefunden hat. Deshalb wird im nächsten Schritt anhand einer longitudinalen Mehrebenenanalyse eine differenzierte Untersuchung der Bedeutungsverschiebung des Werts der Konformität vorgenommen. Anhand der longitudinalen Mehrebenenanalyse lässt sich zudem feststellen, ob Effekte der Parteipräferenz auch unter Berücksichtigung von Drittvariablen weiterhin wirksam sind.

Zur Überprüfung der zeitlichen Äquivalenz der Wertemessungen wurde das Wertemodell von Schwartz in Anlehnung an Davidov et al. (2008) aufbereitet und anhand von konfirmatorischen Faktorenanalysen die Messinvarianz zwischen den beiden Erhebungszeitpunkten überprüft.^13^ Tatsächlich gelingt es mit diesem Modell, sowohl für die Daten der VIC Studie 2020 (chi^2^ = 645,4, *p* < 0,001, CFI = 0,90; RMSEA = 0,06) als auch für die Daten der VIC Studie 2021 (chi^2^ = 532,4, *p* < 0,001, CFI = 0,92; RMSEA = 0,05) mittels mehrerer Modifikationen^14^ eine ausreichende Modellgüte zu erzielen. In weiterer Folge wurde die Messinvarianz des Wertemodells über die beiden Zeitpunkte evaluiert. Die Methode der Multi Group Confirmatory Factor Analysis (MGCFA) setzt dabei für eine angemessene Prüfung der Äquivalenz des Konstrukts zumindest drei hierarchische Ebenen der Konstrukt‑, Skalen- und Itemäquivalenz voraus (Byrne und Stewart 2006). In einem ersten Schritt sollte *konfigurale Äquivalenz* erzielt werden. Hierbei wird getestet, ob im Zeitverlauf zumindest dieselben Items mit den jeweiligen Dimensionen in Verbindung stehen. Um mit den verwendeten Konzepten Beziehungen zwischen Variablen errechnen zu können, muss zumindest *metrische Äquivalenz* vorliegen. Das bedeutet, dass nicht nur dieselben Items eine Faktorenstruktur bilden, sondern dass auch die Korrelationen der Items mit den jeweiligen Faktoren (die Faktorladungen) über die beiden Zeitpunkte weitgehend ident sind. Der dritte und zugleich letzte Schritt, die Prüfung der *skalaren Äquivalenz*, verlangt die inhaltliche Gleichwertigkeit der Items, die für den Mittelwertsvergleich über die Zeit nötig ist. Dabei werden die Item-Intercepts für alle Gruppen als gleich definiert. Bei ungleichen Item-Intercepts verdeutlicht der Skalenwert schließlich nicht einen „wahren Unterschied“ auf der jeweiligen Dimension, sondern eine zeitlich spezifische Reaktion auf ein manifestes Item.

Bei Äquivalenztestungen wird empfohlen, primär auf den CFI-Wert und den RMSEA-Wert Bezug zu nehmen. Nach Cheung und Rensvold (2002) ist die nächsthöhere Äquivalenzstufe dann bestätigt, wenn der Abfall des CFI-Wertes geringer als 0,01 ausfällt (ebd., S. 251). Tab. 1 zeigt, dass es mittels der Methode der MGCFA über die beiden Messzeitpunkte gelingt, *konfigurale, metrische und skalare Äquivalenz* zu erzielen. Der CFI-Wert bleibt bei Gleichsetzung der Intercepts auf ähnlichem Niveau und auch der RMSEA-Wert deutet auf eine hohe Modellgüte hin. Damit sind die Voraussetzungen für die Durchführung der Mittelwertsvergleiche in den Wertedimensionen über die beiden Zeitpunkte der VIC-Studie hinreichend erfüllt, sodass Aussagen über die Verschiebungen von Wertprioritäten anhand der vorliegenden Paneldaten getroffen werden können.

**Table 1:** 

		*Χ*^*2*^	*df*	*p des Modells*	*CFI*	*RMSEA*
VIC 1 vs. VIC 2	Konfigural	1264,784	367	< 0,000	0,909	0,035
	Metrisch	1294,520	384	< 0,000	0,908	0,035
	Skalar	1349,697	405	< 0,000	0,904	0,034

## Empirische Ergebnisse zu den Verschiebungen von Wertprioritäten

### Werteverschiebungen im Verlauf der Pandemie

**Figure 2:**
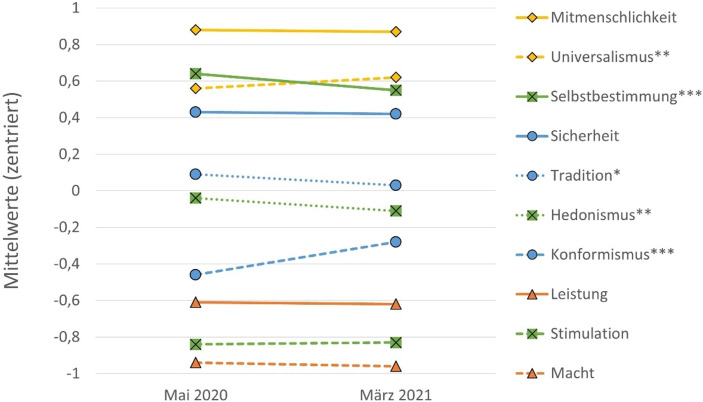

Abb. 2 zeigt, wie sich die Bedeutung der zehn Grundwerte im Verlauf der Pandemie – von Mai 2020 bis März/April 2021 – in der Panelstichprobe (*N* = 995) entwickelt hat. Statistisch signifikante Verschiebungen von Wertprioritäten werden in der Abbildung mit Sternchen gekennzeichnet.^15^ Insgesamt weisen diese ersten Befunde sowohl auf eine deutliche Stabilität als auch auf Verschiebungen einzelner Wertprioritäten in der österreichischen Bevölkerung hin (siehe Abb. 2). Die Werte der Mitmenschlichkeit und Sicherheit sowie jene der Leistung, Stimulation und Macht weisen innerhalb des ersten Jahres der Pandemie eine hohe Kontinuität auf. Überraschend ist hier vor allem, dass der Stellenwert von Sicherheit sowie der Wert der Stimulation in Zeiten eingeschränkter Möglichkeiten zur Selbstverwirklichung und Freizeitgestaltung unverändert blieb. Aus methodenkritischer Sicht muss jedoch auch auf die Itemschwierigkeit der Fragen zu Mitmenschlichkeit und Leistung hingewiesen werden. „Es ist wichtig, sich für Menschen einzusetzen, denen man nahesteht“ als Indikator für den Wert Mitmenschlichkeit findet eine derart große Zustimmung, dass der prognostizierte Anstieg von Solidarität im Kontext der Corona-Krise womöglich nicht adäquat erfasst werden kann. Analog dürfte die starke Ablehnung von Leistung auch auf die Frageformulierung „es ist wichtig, seine Fähigkeiten zu zeigen und von anderen bewundert zu werden“ zurückzuführen sein.

Auch wenn Grundwerte als stabile Elemente im System der Persönlichkeit und als wesentliche kulturelle Marker der Gesellschaft gelten (Schwartz 2006), so zeigen sich doch einige statistisch signifikante Verschiebungen von Wertprioritäten in der österreichischen Gesellschaft. Selbstbestimmung und Hedonismus verlieren etwa an Bedeutung, während der Stellenwert des Werts der Konformität wächst (Selbstbestimmung ist allerdings nach wie vor wichtiger als Konformität). Entgegen dem Trend zur Konformität verliert Tradition – die in der ersten Phase der Pandemie im Vergleich zum Vorkrisenniveau eine Renaissance erfahren hat (Aschauer et al. 2022) – im Verlauf der Pandemie geringfügig an Bedeutung. Der Wert des Universalismus (Engagement für Gleichberechtigung, Toleranz, Umweltschutz) wird für die Befragten hingegen etwas wichtiger. Insgesamt ist den Befragten Universalismus, Selbstbestimmung, Mitmenschlichkeit und Sicherheit allerdings nach wie vor am wichtigsten. Mitmenschlichkeit wird zu beiden Erhebungszeitpunkten konstant mit der höchsten Wichtigkeit versehen.

Fasst man die Veränderungen im Sinne der vier übergeordneten Dimensionen bzw. zwei Achsen von Werthaltungen von Schwartz (siehe Abb. 1) zusammen, so zeigt sich in Hinblick auf die übergeordnete Dimension Statuserhöhung (Macht, Leistung) versus Selbst-Transzendenz (Mitmenschlichkeit, Universalismus) keine signifikante Veränderung, während jedoch eine statistisch signifikante Verschiebung von Offenheit für Veränderung zu Bewahrung der Ordnung (Konservatismus) erkennbar wird.^16^ Für diesen Trend verantwortlich ist eine abnehmende Bedeutung der Werte Selbstbestimmung und Hedonismus sowie eine Zunahme des Werts der Konformität. Bei den anderen Werten dieser Werteachse (Sicherheit, Stimulation und Tradition) lassen sich hingegen keine Veränderungen feststellen. Die Bedeutung von Tradition nimmt sogar etwas ab, jene der Sicherheit bleibt stabil. Wesentlicher Teil des bereits in früheren Studien beobachteten Konservatismus-Trends (siehe Abschn. 2.1) ist also ein Konformitätstrend, der sich in den Wertvorstellungen innerhalb eines Jahres Pandemieerfahrung erkennen lässt.

### Verschiebung der Wertprioritäten innerhalb von Wähler*innengruppen

In weiterer Folge wird überprüft, ob die Verschiebung von Wertprioritäten auf der Achse Offenheit für Veränderung versus Bewahrung der Ordnung in allen Wähler*innengruppen gleichermaßen auftritt. Vor dem Hintergrund der unterschiedlichen *party cues* rund um die von der Regierung verhängten Schutzmaßnahmen, der Anti-Establishment Attitüde der FPÖ und der „corona-spezifischen“ Bedeutung einzelner Items der Schwartz-Werteskala sind durchaus Unterschiede im beobachteten Konformitätstrend erwartbar. Um dies näher zu untersuchen, werden die Schwartz-Werte aus der ersten Welle der VIC-Studie (Mai 2020) mit jenen aus der zweiten Welle (März/April 2021) pro Partei verglichen. Berücksichtigt werden nur Befragte, die bei der Frage nach ihrer aktuellen Parteipräferenz in der zweiten Welle dieselbe Partei angaben, die sie auch bei der letzten Nationalratswahl gewählt haben. Diese Vorgehensweise dient dazu, etwaigen Verzerrungen durch Wähler*innenwanderungen zwischen den Parteien vorzubeugen.

**Figure 3:**
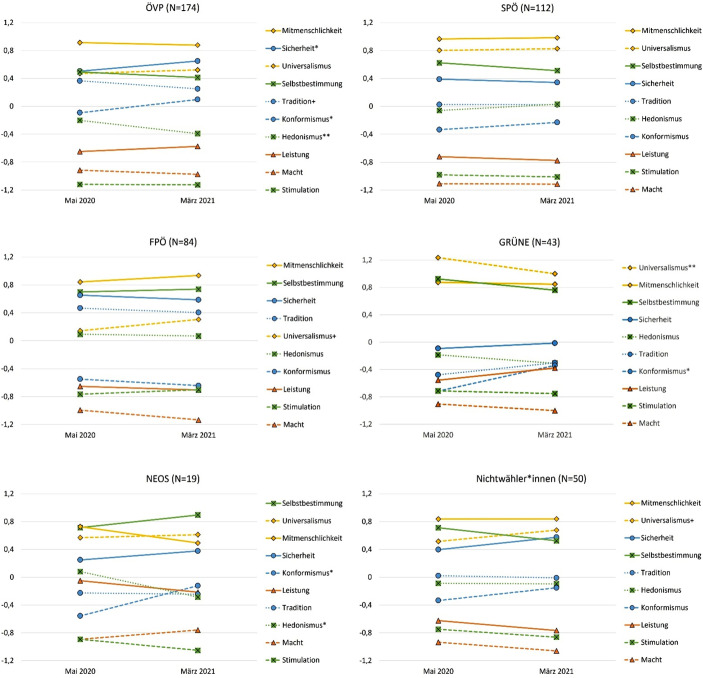

Die Ergebnisse in Abb. 3 bestätigen auch für die einzelnen Wähler*innengruppen eine stark ausgeprägte Kontinuität der Wertorientierungen nach Schwartz, die sich im Zuge der COVID-19-Pandemie fortsetzt. Besonders stabil sind die Werte der Wähler*innen der Oppositionsparteien SPÖ und FPÖ und jene der Nicht-Wähler*innen. Der insgesamt konstatierte Konformitätstrend lässt sich vor allem bei Wähler*innen der beiden Regierungsparteien ÖVP und Grüne beobachten, was ein Indiz dafür ist, dass die *cues* der Parteien zur Befolgung der Corona-Schutzmaßnahmen ihre Wirkung erzielt haben dürften. Die Ergebnisse der NEOS-Wähler*innen lassen sich aufgrund der geringen Fallzahl (19 Personen) hinsichtlich statistisch signifikanter Veränderungen in den Wertorientierungen nicht interpretieren.

Überraschend ist, dass der Wert der Sicherheit ausschließlich für Wähler*innen der ÖVP an Bedeutung gewinnt und der Wert des Universalismus einzig für die Wähler*innen der Grünen etwas an Stellenwert verliert. Dabei muss jedoch festgehalten werden, dass Sicherheit für die ÖVP-Wähler*innen und Universalismus für die Grün-Wähler*innen^17^ zu beiden Zeitpunkten einen besonders hohen Stellenwert einnimmt. Generell werden altruistische Werte (wie Mitmenschlichkeit) quer durch alle Wähler*innengruppen mit höchster Wichtigkeit versehen.

Diametral zum partiellen Konformitätstrend verliert innerhalb des ersten Jahres der Pandemie der Wert des Hedonismus für Wähler*innen der ÖVP an Bedeutung. Werte, die auf Leistungsbereitschaft abzielen, sind für Grün-Wähler*innen von vergleichsweise großer Bedeutung; die Werthaltung des Machtstrebens rangiert bei nahezu allen Wähler*innengruppen an letzter Stelle.

Verschiebungen in den Wertprioritäten könnten auch mit Veränderungen in der Wähler*innenschaft an sich zusammenhängen, zumal im Zuge der zweiten Erhebung sowohl das Wahlverhalten bei der letzten Nationalratswahl 2019 als auch die aktuelle Parteipräferenz abgefragt wurde. Die Ergebnisse zeigen hierzu, dass die Wähler*innenspektren der ÖVP, SPÖ und FPÖ einigermaßen stabil sind; 70 % (ÖVP) bis knapp 85 % (SPÖ) würden der zuletzt gewählten Partei treu bleiben. Für die Kleinparteien zeigt sich zum Befragungszeitpunkt hingegen ein anderes Bild: Bei den Grünen waren im März/April 2021 nur noch rund 45 % bereit, die Partei wieder zu wählen.^18^ Auffallend ist zudem, dass Nichtwähler*innen am stärksten zur FPÖ tendieren.^19^

### Vertiefende Analysen zum Konformitätstrend in der Corona-Pandemie

In einem letzten Schritt der empirischen Auswertung soll nun der in den vorangegangenen Analysen beobachtete Konformitätstrend mit der Methode einer longitudinalen Mehrebenenanalyse weiter untersucht werden. Mit der Methode der longitudinalen Mehrebenenanalyse können simultan Veränderungen der Wertprioritäten derselben Individuen von Mai 2020 bis März/April 2021 gemessen und der Einfluss von wesentlichen zeit- und personenbezogenen Prädiktoren auf Konformität geschätzt werden. Die hierarchische Struktur der Daten umfasst dabei die Zeitebene sowie zeitabhängige (auf Ebene 1) und zeitunabhängige Prädiktoren (Ebene 2) auf der Personenebene.

Die Werteverschiebungen werden anhand von vier Modellen untersucht (vgl. zusammenfassend Hox 2010, S. 54–59). Im ersten Modell (Nullmodell) wird ausschließlich der Zeiteffekt (ohne Prädiktoren) berechnet. Im zweiten Modell (Prädiktorenmodell) wird zunächst der für die Analyse zentrale Erklärungsfaktor der Parteipräferenz aufgenommen (in Modell 2a als zeitunabhängiger Prädiktor und in Modell 2b als zeitabhängiger Prädiktor). Um den Effekt der Parteipräferenz unter Kontrolle von Drittvariablen zu untersuchen, werden in einem dritten Modell die Bildung, das Geschlecht und das Alter (personenbezogene Prädiktoren) sowie die politische Links-Rechts-Orientierung (als zeitabhängige Prädiktoren) kontrolliert. Im vierten Modell werden schließlich zusätzliche Interaktionseffekte inkludiert, um festzustellen, ob es im Zeitverlauf je nach Parteipräferenz sowie nach Alter und Geschlecht zu Werteverschiebungen kommt. Die Varianzaufklärung in den Modellen wird nicht nur auf der Zeit- und Personenebene (Ebene 1 und 2) berichtet, sondern es wird gezeigt, welche Erklärungskraft die Interaktionseffekte über die Zeit besitzen (Fairbrother 2014).

Aus Modell 1 in Tab. 2 wird ersichtlich, dass die Wichtigkeit der Wertorientierung der Konformität über die Zeit (mittlere Veränderung = 0,18) höchstsignifikant angestiegen ist. Der Intraklassenkoeffizient von 0,44 besagt, dass knapp die Hälfte der Varianz auf Unterschiede zwischen Personen zurückzuführen ist, während rund 56 % der Varianz den intraindividuellen Veränderungen über die Zeit geschuldet sind. Berücksichtigt man die Unterschiede zwischen den Wähler*innengruppen in Modell 2, wird zunächst deutlich, dass Konformität für die Wähler*innen der ÖVP eine größere Bedeutung hat als für Wähler*innen der Grünen, der FPÖ und „anderer Parteien“. Dieses Muster bestätigt sich auch, wenn die Parteipräferenz als zeitabhängiger Prädiktor in das Modell aufgenommen wird (siehe Modell 2b), auch wenn die Unterschiede hier geringer ausfallen als in Modell 2a.^20^

**Table 2:** 

			Modell 1: Nullmodell inklusive Zeit	Modell 2a: Zeit & Parteipräferenz zeitunabhängig	Modell 2b: Zeit und Parteipräferenz zeitabhängig	Modell 3: Prädiktoren Personenebene	Modell 4: Prädiktoren Zeit- & Personenebene & Interaktion mit Zeiteffekt (Wählerpräferenz auf Basis NR 2019)
–	Intercept		−0,46	−0,27	–	−0,42	−0,41
Zeiteffekt	Veränderung t1–t2		0,18***	0,18***	–	0,20**	0,15*
Personenbezogene Prädiktoren	Parteipräferenz (Referenz: ÖVP)	SPÖ	–	−0,14	−0,08	−0,14	−0,17^+^
		FPÖ	–	−0,35***	−0,25**	−0,30*	−0,20^+^
		GRÜNE	–	−0,40***	−0,24**	−0,26*	−0,32**
		NEOS^a^	–	(−0,34*)	(−0,30**)	(−0,19)	(−0,27^+^)
		Andere Parteien	–	−0,58**	−0,46***	−0,47*	−0,72**
		Nichtwähler*innen	–	−0,13	−0,01	−0,04	0,01
	Ausbildung (Referenz: Master)	Pflichtschule	–	–	–	0,25*	0,26*
		Abgeschlossene Lehre	–	–	–	0,12	0,13
		Mittlere Schule	–	–	–	0,05	0,07
		AHS/BHS	–	–	–	0,06	0,06
		Bachelor	–	–	–	0,02	0,03
	Geschlecht		–	–	–	−0,07	−0,10
	Geburtsjahr		–	–	–	−0,01*	−0,01***
	Links-Rechts-Orientierung		–	–	–	0,02	0,02
Interaktionseffekte mit Zeit	Zeit * Geschlecht		–	–	–	–	0,06
	Zeit * Geburtsjahr		–	–	–	–	0,00
	Zeit * SPÖ		–	–	–	–	0,07
	Zeit * FPÖ		–	–	–	–	−0,23*
	Zeit * NEOS^a^		–	–	–	–	(0,15)
	Zeit * Grüne		–	–	–	–	0,17
	Zeit * Andere Parteien		–	–	–	–	0,54*
	Zeit * Nichtwähler*innen		–	–	–	–	−0,10
Varianzkomponenten	Level 1 (Zeitebene)		0,46	0,44	0,46	0,40	0,39
	Level 2 (Personenebene)		0,59	0,59	0,55	0,56	0,56
	Slope-Varianz (Zeiteffekt)		–	–	–	0,06	0,06
Varianzaufklärung	Level 1 (Zeitebene)		–	4,3 %	0,0 %	13,0 %	15,2 %
	Level 2 (Personenebene)		–	0,0 %	3,5 %	5,1 %	5,1 %
	Slope-Varianz (Zeiteffekt)		–	–	–	–	3,8 %
	ICC		0,438	–	–	–	–
Devianz	–		5360,6	4547,6	5329,78	4394,7	4372,9
–	Anzahl geschätzter Parameter		4	10	10	19	27
–	Stichprobe (ungewichtet)		995/t = 2	853/t2	995/t = 2	848/t = 2	848/t2

Statistische Signifikanz: ^+^*p* < 0,10; *p < 0,05; **p < 0,01; ***p < 0,001

*NR* Nationalratswahl

^a^Aufgrund der geringen Fallzahl können die Werte für NEOS-Wähler*innen nicht interpretiert werden

Kontrolliert man die Unterschiede zwischen den Wähler*innengruppen nach Bildung, Alter und Geschlecht (siehe Modell 3), nehmen die Unterschiede zur ÖVP ab, es zeigen sich jedoch nach wie vor deutlich niedrigere Konformitätswerte für die Wähler*innen der FPÖ, der Grünen und „anderer Parteien“. Der Stellenwert von Konformität ist zudem für Pflichtschulabsolvent*innen und ältere Personen höher. Alle aufgenommenen Variablen in Modell 3 sind in der Lage, rund 5 % der Varianz auf Personenebene aufzuklären. Die Interaktionseffekte über die Zeit zeigen (siehe Modell 4 in Tab. 2), dass sich FPÖ-Wähler*innen im Zeitverlauf vom Wert der Konformität eher abwenden, während dieser Wert für die Wähler*innen der Restgruppe der „anderen Parteien“ wichtiger wird. Die aufgenommenen Variablen erklären rund 24 % der sogenannten Slope-Varianz, d. h. sie haben wesentlichen Einfluss auf die (unterschiedlichen) Schwankungen des Konformitätswerts.

Die Ergebnisse der Mehrebenenanalyse legen somit nahe, dass die Wertorientierung der Konformität im Zuge der Corona-Krise vor allem für die FPÖ-Wähler*innen nicht an Bedeutung gewinnt. Von Interesse ist, dass sich zwischen den Wähler*innen der beiden Regierungsparteien keine wachsende Diskrepanz abzeichnet.

## Resümee

In diesem Beitrag wurde untersucht, inwieweit es in Zeiten drastischer gesellschaftlicher Einschnitte und Herausforderungen durch die COVID-19-Pandemie zu einer Verschiebung der Wertprioritäten in der österreichischen Gesellschaft kam. Die Analyse erfolgte anhand der Werteskala von Shalom Schwartz, die in den letzten Jahrzehnten in verschiedenen nationalen und international vergleichenden Umfragen eingesetzt und in Österreich in der ersten und zweiten Welle der Values-in-Crisis-Panelstudie (VIC) erhoben wurde.

Als erstes zentrales Ergebnis können wir festhalten, dass sich die Wichtigkeit der zehn Grundwerte im Vergleich der beiden VIC-Studien von Mai 2020 bis März/April 2021 nur geringfügig geändert hat. Die Wertorientierungen der österreichischen Bevölkerung erweisen sich damit entgegen aktuellen Zeitdiagnosen, die einen tiefgreifenden Wertewandel durch die Erschütterungen des sozialen Lebens in der Corona-Pandemie konstatieren, als durchaus stabil. Dieser Befund einer weitgehenden Kontinuität grundlegender Wertorientierungen entspricht auch der Grundkonzeption des Wertekonzepts von Schwartz (1992, 2006). Die Stabilität lässt sich wie folgt charakterisieren: Am wichtigsten ist den Menschen zu beiden Erhebungszeitpunkten der Wert der Mitmenschlichkeit, gefolgt von den Werten des Universalismus, der Selbstbestimmung und Sicherheit. Vergleichsweise gering(er) ist die Bedeutung der Werte Hedonismus, Tradition, Konformität und Leistung. Macht und Stimulation (Wunsch nach Abwechslung) werden jeweils als am wenigsten wichtig eingestuft.

Bei etwa der Hälfte der zehn Grundwerte lassen sich jedoch auch signifikante Mittelwertunterschiede feststellen, die es im Kontext der Corona-Pandemie zu interpretieren gilt. Angesichts der periodisch wiederkehrenden Corona-Infektionswellen und der damit einhergehenden staatlichen Maßnahmen wurde Konformität für einen Teil der Bevölkerung im Vergleichszeitraum wichtiger; auch Universalismus (im Sinn von Chancengleichheit und Verständnis für Menschen, die anders sind als man selbst) wurde nach dem ersten Jahr der Pandemie etwas höher eingestuft. Der Wunsch nach einer hedonistischen Lebensweise verlor hingegen etwas an Bedeutung. Im Einklang mit dem Forschungsstand (Voicu et al. 2016; Aschauer et al. 2022) lässt sich also eine Verschiebung auf der Werteachse vom Pol Offenheit für Neues zum Pol Bewahren des Bestehenden (Konservatismus) beobachten. Diese Verschiebung zeigt sich insbesondere in einer verstärkten Wichtigkeit der Werthaltung Konformität, deren Bedeutung sich im Kontext der Corona-Pandemie hin zur Befolgung von staatlich verordneten Schutzmaßnahmen veränderte. Die „corona-spezifische“ Bedeutung von Konformität zeigt sich auch bei der Betrachtung der Werteprofile der Wähler*innen: Ein Konformitätstrend lässt sich nämlich insbesondere bei den Wähler*innen der Regierungsparteien ÖVP und Grüne beobachten, während sich dieser einzig bei den Wähler*innen der FPÖ nicht zeigt.

Im letzten Teil der Analyse wurde schließlich der Wert der Konformität, dessen Wichtigkeit sich im Verlauf der Corona-Pandemie am deutlichsten geändert hat, im Rahmen einer multivariaten Analyse untersucht. Die Ergebnisse zeigen, dass Konformität für die Wähler*innen der Regierungspartei ÖVP von Beginn der Corona-Pandemie an wichtiger war als für Wähler*innen anderer Parteien. Für Grün-Wähler*innen hat Konformität traditionell einen geringeren Stellenwert; bereits in der ersten Phase der Pandemie im Mai 2020 war Konformität wichtiger für Grün-Wähler*innen als für FPÖ- und NEOS-Wähler*innen. Im Verlauf der Pandemie setzte sich dieser Trend bei den Grün-Wähler*innen, die ihrer Partei treu blieben, tendenziell fort.

Nun sind Universalismus und Selbstbestimmung für Grün-Wähler*innen traditionell wichtige Werte. Mit dem Eintritt in die Regierung, kurz vor Beginn der Pandemie, sprachen sich jedoch auch die Grünen deutlich für eine Einschränkung der Selbstbestimmung aus, da sie den Schutz der Gesundheit und die Rücksichtnahme auf gesundheitlich gefährdete Gruppen zur höchsten Maxime erhoben. Letzteres dürfte auch ihrer im Vergleich zu anderen Wähler*innen ausgeprägten universalistischen Überzeugung entspringen. Auch in diesem Kontext spielen *party cues* eine wichtige Rolle, schließlich appellierte der grüne Gesundheitsminister häufig für die Einhaltung der Corona-Regeln im Sinne der Solidarität mit den Hochrisikogruppen.

Bei der FPÖ ist die Tendenz umgekehrt. Nach dem Ende der Regierungsbeteiligung und mit dem Beginn der Corona-Pandemie schlug die FPÖ einen fundamentalen Oppositionskurs ein und übte in einer Anti-Establishment Attitüde (z. B. Crouch 2021) in zunehmendem Maße Kritik an den Corona-Maßnahmen der Regierung. Indem die FPÖ zum Sprachrohr für Gegner*innen der Corona-Maßnahmen der Regierung wurde, lehnten ihre Sympathisant*innen Konformität im Verlauf der Corona-Pandemie ab. Non-konformes Verhalten dürfte sich für die befragten FPÖ-Wähler*innen somit auch auf die Ablehnung der Corona-Regeln der Regierung beziehen. Sicherlich spielen auch hier *cues* eine zentrale Rolle, die die FPÖ an ihre Unterstützer*innen gesandt hat (Heinisch 2004, S. 252 ff.; Spier 2006, S. 37 f.).

Unsere Analysen zeigen, dass im Zeitraum von Mai 2020 bis März/April 2021 geringfügige Verschiebungen der Wertprioritäten vorwiegend bei jenen Werte-Items auftreten, deren Formulierung im Kontext der Pandemie eine spezifische Konnotation bekommen. Dies betrifft in erster Linie die drei Werte Konformität, Selbstbestimmung und Hedonismus. Im Kontext der Pandemie finden es viele Menschen wichtig, „sich an Regeln zu halten, selbst wenn es niemand sieht“ (Konformismus). „Selbst zu entscheiden, was man tut“ (Selbstbestimmung) und „Dinge zu tun, die einem Vergnügen bereiten“ (Hedonismus) wird hingegen im Kontext der Pandemie als weniger wichtig beurteilt. Bei Itemformulierungen, die keinen spezifischen Bezug zur Pandemie aufweisen – wie etwa „Sitten und Gebräuche zu befolgen, die durch Familie und Religion überliefert wurden“ (Tradition) oder „seine Fähigkeiten unter Beweis zu stellen und bewundert zu werden“ (Leistung) – lassen sich hingegen kaum Änderungen im Zeitverlauf feststellen. Daher bleibt abzuwarten, ob die von uns beobachteten Verschiebungen von Wertprioritäten nach dem Ende der Pandemie fortbestehen werden. Die Tatsache, dass Veränderungen vor allem bei den „pandemie-sensiblen“ Wertedimensionen festgestellt wurden und dass diese Veränderungen durch die Positionen der politischen Parteien in Hinblick auf die Pandemie-Maßnahmen beeinflusst zu sein scheinen, legen die Vermutung nahe, dass es sich eher um kurzfristige Reaktionen auf die Krise, und nicht um einen langfristigen Wertewandel handeln dürfte.

## Anhang

**Table 3:**

Erhebung	
Hier beschreiben wir kurz einige Personen. Bitte hören Sie sich die einzelnen Beschreibungen genau an und sagen Sie mir bitte, wie sehr Ihnen jede dieser Personen gleicht oder nicht gleicht. Die zur Verfügung stehenden Antwortkategorien lauten „gleicht mir etwas“, „gleicht mir wenig“, „gleicht mir nicht“ und „gleicht mir ganz und gar nicht“.	
*Selbstbestimmung*	Es ist ihm/ihr wichtig, neue Ideen zu entwickeln und kreativ zu sein. Er/Sie unternimmt alles gerne auf seine/ihre eigene Art und Weise.
	Es ist ihm/ihr wichtig, selbst zu entscheiden, was er/sie tut. Er/Sie ist gerne frei und unabhängig von anderen.
*Macht*	Reichtum ist ihm/ihr wichtig. Er/Sie möchte viel Geld und Luxusgüter besitzen.
	Es ist ihm/ihr wichtig, von anderen respektiert zu werden. Er/Sie möchte, dass die Leute das tun, was er/sie sagt.
*Universalismus*	Für ihn/sie ist es wichtig, dass jeder Mensch auf dieser Welt gleichbehandelt wird. Er/sie glaubt, dass jeder Mensch die gleichen Chancen im Leben haben sollte.
	Es ist ihm/ihr wichtig, Leuten zuzuhören, die von ihm/ihr verschieden sind. Auch wenn er/sie mit ihnen nicht übereinstimmt, möchte er/sie sie trotzdem verstehen.
	Er/Sie ist überzeugt, dass sich die Menschen um ihre Umwelt kümmern sollen. Umweltschutz ist ihm/ihr wichtig.
*Leistung*	Es ist ihm/ihr wichtig, seine/ihre Fähigkeiten unter Beweis zu stellen. Er/sie möchte, dass ihn/sie Leute für das bewundern, was er/sie tut.
	Es ist ihm/ihr wichtig, sehr erfolgreich zu sein. Er/sie hofft, dass andere Leute seine/ihre Leistungen anerkennen.
*Sicherheit*	Es ist ihm/ihr wichtig, in einer sicheren Umgebung zu leben. Er/Sie vermeidet alles, das seine/ihre Sicherheit gefährden könnte.
	Es ist ihm/ihr wichtig, dass der Staat seine Sicherheit gegen alle Bedrohungen wahrt. Er/Sie möchte einen starken Staat, der seine Bürger/innen beschützen kann.
*Stimulation*	Er/Sie liebt Überraschungen und sucht immer nach Neuem. Für ihn/sie ist es wichtig, im Leben möglichst viele verschiedene Dinge zu unternehmen.
	Er/Sie ist auf Abenteuer aus und nimmt dafür Risiken auf sich. Er/Sie möchte ein aufregendes Leben führen.
*Konformität*	Er/Sie glaubt, dass Leute das machen sollten, was man ihnen sagt. Er/Sie meint, dass Leute sich immer und überall an Regeln halten sollten, selbst wenn es niemand sieht.
	Es ist ihm/ihr wichtig, ein anständiges Leben zu führen. Er/Sie möchte alles vermeiden, was Leute als Fehltritt bezeichnen könnten.
*Tradition*	Es ist ihm/ihr wichtig, zurückhaltend und bescheiden zu sein. Er/Sie versucht, keine Aufmerksamkeit auf sich zu lenken.
	Traditionen sind ihm/ihr wichtig. Er/Sie möchte jene Sitten und Gebräuche befolgen, die ihm/ihr durch Religion oder die Familie überliefert wurden.
*Hedonismus*	Spaß zu haben, ist ihm/ihr wichtig. Er/Sie verwöhnt sich gerne.
	Er/Sie nutzt jede Gelegenheit, um Spaß zu haben. Es ist ihm/ihr wichtig, Dinge zu tun, die ihm/ihr Vergnügen bereiten.
*Mitmenschlichkeit*	Es ist ihm/ihr wichtig, den Menschen in seiner/ihrer Umgebung zu helfen. Er/Sie möchte sich um deren Wohlergehen sorgen.
	Ihm/Ihr ist es wichtig, gegenüber seinen/ihren Freunden treu zu sein. Er/Sie möchte für die Menschen da sein, die ihm/ihr nahestehen.

## Footnotes

1 Heinz Bude im Interview beim Tagesspiegel (https://www.tagesspiegel.de/kultur/verwundbarkeit-macht-solidarisch-4696723.html).

2 Jutta Allmendinger im Interview bei Die Zeit (2022).

3 Vgl. hier z. B. den Gastkommentar der Neuen Züricher Zeitung zur Situation in Österreich am 07.01.2021 (Mijnssen 2021).

4 Laut Befragungen im Rahmen des Austrian Corona Panel Projects ist von März 2020 bis April 2021 vor allem das Vertrauen in das Parlament, in die Regierung und in das öffentlich-rechtliche Fernsehen gesunken (Krejka et al. 2021).

5 Mit dem Begriff des „nostalgischen Pessimismus“ meint Crouch eine Geisteshaltung der Sehnsucht nach Vergangenem, nach welcher die „goldene Vergangenheit“ durch eine „Invasion von außen“ regelrecht zerstört worden wäre (Crouch 2021, S. 144 ff.; über Österreich S. 159).

6 Empirische Bestätigung dieser *partisan cues* finden sich in zahlreichen experimentellen Studien (z. B. Bakker et al. 2020 oder Barber und Pope 2019).

7 Hinzu kommt, dass sich Individuen vermehrt Informationen von außerparteilichen Akteur*innen z. B. aus sozialen Medien beschaffen. Hierbei spielt *selective exposure* eine Rolle – also die Tendenz, dass Menschen jene Informationsquellen wählen, die ihre eigenen Überzeugungen eher bestätigen (Iyengar und Hahn 2009, S. 20 ff.). Das kann in Folge zur Verbreitung von Falschinformationen führen (Burton und Koehorst 2020, S. 2), den sogenannten Echo-Kammer-Effekt (Rau und Stier 2019) verstärken und letztlich zur politischen Polarisierung beitragen (Stroud 2010, S. 566 ff.).

8 Eine Regierungsbeteiligung der Grünen war möglich, da bei der letzten Wahl 2019 die Problematik der Klimakrise breite Resonanz erfuhr und auch Bevölkerungsgruppen über die Kernwähler*innenschaft hinaus angesprochen werden konnten.

9 Die Befragung deckt sich in Bezug auf Geschlecht, Alter, Bildung und Region weitgehend mit den Daten der Statistik Austria. Dennoch dürften online-affine Personen eher an der Studie teilgenommen haben, weshalb insbesondere im höheren Alters- und niedrigen Bildungsbereich leichte Verzerrungen der Stichprobe zu erwarten sind.

10 Es wurden nur Befragte berücksichtigt, die für die Schwartz-Skala mehr als 100 s Ausfülldauer benötigt haben. Wir haben hier bewusst in der Umfrage einen Zeitstempel gesetzt, um Tendenzen des „Satisficing“ in längeren Umfragen entsprechend zu kontrollieren (siehe z. B. Krosnick 1991). Die ausgeschlossenen Befragten sind im Mittel rund fünf Jahre jünger als die inkludierten und weisen auch niedrigere Ausbildungsniveaus auf. Die ausgeschlossene Gruppe unterscheidet sich jedoch weder hinsichtlich der Verteilung nach Geschlecht oder Parteipräferenz signifikant von der analysierten Gruppe.

11 Im Wortlaut wird gefragt: „Im Folgenden sehen Sie Beschreibungen einiger Personen. Bitte schauen Sie sich jede Beschreibung an und geben Sie an, wie ähnlich oder unähnlich Ihnen die jeweils beschriebene Person ist.“.

12 Die Werteangaben werden dabei rund um den Mittelwert zu allen Werteangaben zentriert. Diese Vorgehensweise wird von Schwartz empfohlen, um starke Zustimmungstendenzen zu reduzieren (Schwartz et al. 2015, S. 4 f.).

13 Aus Platzgründen können die Modelle hier nicht dargestellt werden. Die einzelnen Berechnungen, die über AMOS durchgeführt wurden, können bei den Verfasser*innen des Artikels angefordert werden.

14 Es mussten einzelne Korrelationen zwischen Fehlertermen sowie einzelne Querladungen zu Items anderer Werthaltungen eingeführt werden, um die Modellqualität zu erhöhen. Auch hier erfolgte das Vorgehen weitgehend analog zu den Analysen von Davidov et al. (2008, S. 434).

15 Insgesamt zeigt sich auch ein hoher Grad der Übereinstimmung der Beantwortung der Schwartz-Werte über die Zeit. Die Korrelation zwischen den Antworten der Befragten im Mai 2020 und März/April 2021 beträgt bei den einzelnen Items zwischen 0,40 und 0,55. Zur intertemporalen Stabilität der Schwartz-Werteskala siehe auch Vecchione et al. (2016).

16 Dies bestätigen auch die Ergebnisse der Analyse der übergeordneten Werteachsen, die aus Platzgründen im vorliegenden Beitrag nicht dargestellt werden.

17 Dies ist sicherlich auch deshalb der Fall, weil in der Wertedimension Universalismus ein Item zur Wichtigkeit von Umwelt integriert ist (siehe hierzu Tab. 3 im Anhang).

18 Rund 25 % der Grünwähler*innen sympathisieren im März/April 2021 mit der SPÖ, rund 15 % tendieren zu NEOS. Eine detaillierte Analyse muss aus Platzgründen ausgespart werden, kann jedoch bei den Autor*innen angefordert werden.

19 Der FPÖ dürfte es demnach am ehesten gelingen, neue Wähler*innenschichten anzusprechen. Bereits im Juli 2021 gaben bei der Sonntagsfrage wieder zwischen 16 und 18 % der Befragten an, dass sie bei der nächsten Nationalratswahl FPÖ wählen würden (siehe Österreich – Sonntagsfrage Nationalratswahl 2021 | Statista, letzter Zugriff 11.10.2021).

20 Dies ist ein Hinweis darauf, dass sich Werteverschiebungen in Hinblick auf Konformität – unter der Berücksichtigung von Wähler*innenwanderungen – innerhalb der Wähler*innensegmente leicht unterschiedlich manifestieren.
